# Role of 25-Hydroxyvitamin D_3_ and 1,25-Dihydroxyvitamin D_3_ in Chicken Embryo Osteogenesis, Adipogenesis, Myogenesis, and Vitamin D_3_ Metabolism

**DOI:** 10.3389/fphys.2021.637629

**Published:** 2021-02-01

**Authors:** Chongxiao Chen, Dima Lynn White, Brett Marshall, Woo Kyun Kim

**Affiliations:** ^1^Prestage Department of Poultry Science, North Carolina State University, Raleigh, NC, United States; ^2^Department of Poultry Science, University of Georgia, Athens, GA, United States

**Keywords:** vitamin D_3_, chicken embryo, osteogenesis, adipogenesis, myogenesis, metabolism, gene expression

## Abstract

A study was conducted to understand the effects of 25-hydroxyvitamin D_3_ (25OHD) and 1,25-dihydroxyvitamin D_3_ (1,25OHD) administration on the expression of key genes related to osteogenesis, adipogenesis, myogenesis, and vitamin D_3_ metabolism in the chicken embryo. A total of 120 fertilized Cobb 500 eggs were used in the current study and were reared under standard incubation conditions. On embryonic day 3 (ED 3), PBS (C), PBS with 40ng 1,25OHD (1,25D-L), 200ng 1,25OHD (1,25D-H), 40ng 25OHD (25D-L), or 200ng 25OHD (25D-H) were injected into the dorsal vein of developing embryos. Whole embryos were harvested at 1, 3, and 6h post-injection for gene expression analyses (*n*=8). Gene expression for key osteogenesis markers (*RUNX2*: runt-related transcription factor 2; *BMP2*: bone morphogenetic protein 2; *COL1A2*: collagen type I alpha 2 chain; *BGLAP*: bone gamma-carboxyglutamate protein; SPP1: secreted phosphoprotein 1; and *ALP*: alkaline phosphatese), adipogenesis markers (*PPAR-γ*: peroxisome proliferator-activated receptor gamma; *FASN*: fatty acid synthase; and *FABP4*: fatty acid binding protein 4), myogenesis markers (*MYOG*: myogenin; *MYOD1*: myogenic differentiation 1; and *MYF5*: myogenic factor 5), and the enzyme responsible for vitamin D_3_ inactivation (*CYP24A1*: cytochrome P450 family 24 subfamily A member 1) were measured using real-time quantitative reverse transcription polymerase chain reaction (qRT-PCR). Data were normalized by the ΔΔCT method and analyzed using a one-way ANOVA. Results indicated that at 1h post-injection, no differences were found among treatments. At 3h, the early osteogenesis differentiation marker, *ALP*, was increased by 1,25D-H and 25D-H, and 25D-H also stimulated the expression of adipogenesis markers (*FAPB4* and *FASN*). In contrast, the expression of myogenesis markers (*MYOD1* and *MYF5*) was suppressed by 25OHD or 1,25OHD treatments, respectively. At 6h, a late osteogenic differentiation marker, *SPP1*, was increased by 25D-H. *MYOD1* and *MYF5* were continuously suppressed by 25OHD treatments or 1,25D-H. The evidence of vitamin D_3_ metabolite retention was assessed by measuring *CYP24A1* expression. At 1h, there were no differences in *CYP24A1* expression. At 3h, all treatments upregulated *CYP24A1* expression relative to control (PBS) embryos. However, at 6h, only the 25D-H group retained higher *CYP24A1* expression compared to the other treatments. In conclusion, the results suggested both 1,25OHD and 25OHD induced chicken embryo osteogenesis and adipogenesis, but inhibited myogenesis during early chicken embryo development. The higher dosage of 25OHD showed a possibility of a longer retention time in the embryos.

## Introduction

Vitamin D_3_ is essential for normal chicken embryo development (Sunde et al., 1978). It is well-established that Vitamin D_3_ undergoes two biological conversions, first in the liver, to become 25-hydroxyvtamin D_3_ (25OHD) hydroxylated by 25-hydroxylase (*CYP2R1*), and then mainly, in the kidney to become its biologically active form, 1,25-dihydroxyvitamin D_3_ (1,25OHD), which is catalyzed by 1α-hydroxylase (CYP27B1; St-Arnaud, 2008; Christakos et al., 2010). Vitamin D_3_ is primarily stored in the egg yolk in the form of 25OHD to be used by the developing chicken embryo during development (Ovesen et al., 2003; Vieira, 2007; Fatemi et al., 2020). In the poultry industry, 25OHD has become a commercial feed additive for poultry production due to its higher bioactivity than regular vitamin D_3_ (Soares et al., 1995; Atencio et al., 2005a). Considerable research has demonstrated beneficial effects of 25OHD on chicken bone quality (Koreleski and Swiatkiewicz, 2005; Wideman et al., 2015; Chen et al., 2020) and muscle development (Michalczuk et al., 2010; Han et al., 2016).

In addition to utilizing vitamin D_3_ metabolites in poultry feed, the influence of exogenous 25OHD during embryo development has also been studied. The administration of 25OHD can be achieved by *in ovo* injection of 25OHD directly to the yolk of chicken embryos at embryonic day 18 (ED 18), which has shown to reduce late embryo mortality (Fatemi et al., 2020). A less invasive alternative for increasing embryo 25OHD level has been accomplished by providing 25OHD in parent breeder diets. Studies have shown that this practice could significantly improve hatchability and embryo livability (Atencio et al., 2005b; Saunders-Blades and Korver, 2014). However, limited research has been conducted to evaluate the role of vitamin D_3_ metabolites during early development of chicken embryos. Even though considerable cell culture studies have been performed to understand the vitamin D_3_ signaling pathway in osteogenesis, adipogenesis, and myogenesis (Ding et al., 2012; Girgis et al., 2013; van Driel and van Leeuwen, 2014; Dix et al., 2018), the mono-cell culture model fails to account for interactions with alternate cell types or presence of extracellular factors such as fibroblast growth factors, insulin, and growth factors (Rosen and MacDougald, 2006). Osteoblasts, adipocytes, and myoblasts are all differentiated from embryonic stem cells (Kolf et al., 2007). The factors in favor of one cell fate may be at the cost of others (Kolf et al., 2007). As such, it is critical to study the role of vitamin D_3_ in embryogenesis, while all different types of cells are present simultaneously.

Thus, the current study was conducted to understand the role of vitamin D_3_ metabolites on the expression of key osteogenic, adipogenic, myogenic, and vitamin D_3_ metabolism genes in the chicken embryos. Treatments were injected through the dorsal vein of chicken embryo at ED 3. Embryos were then sampled to examine expression of various genes related to osteogenesis, adipogenesis, and myogenesis. The current study provided an overview of how vitamin D_3_ metabolites affect embryogenesis and the metabolism of vitamin D_3_ in the embryo during early developmental stages. The results could also provide insight into the manipulation of nutrients during chicken embryo development and contribute to designing a strategy targeting optimized embryo nutrition.

## Materials and Methods

### Embryo Handling and Treatments

**Figure 1:**
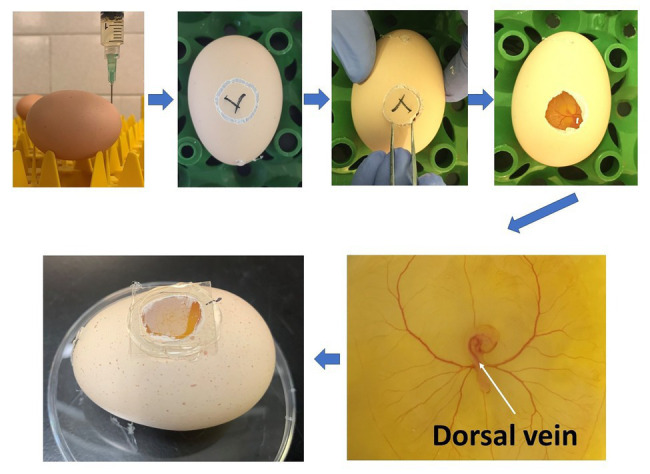
Diagram showing the procedure of *in-ovo* injection at ED 3 and the injection location (white arrow).

The study was approved by the Institutional Animal Care and Use Committee at the University of Georgia and conducted at the research facility of the Department of Poultry Science at the University of Georgia. Cobb 500 fertilized eggs were purchased from Cobb Hatchery (Cleveland, GA, United States). A total of 120 Cobb 500 fertilized eggs (8 eggs/treatment×5 treatments×3 time points) were used in the current study. They were incubated inside a bench incubator (GQF 1502, Savannah, GA, United States) at 37.5°C and around 45% relative humidity. The eggs were positioned horizontally without turning to ensure that the position of embryos was appropriate and consistent for injection. At 72h of incubation, fertilized eggs were sprayed with 70% alcohol, and 1.5ml albumen was carefully taken out from the sharp end of the eggs. The needle hole was sealed with glue immediately to avoid contamination. A mini drill was used for opening a window (around 1cm^2^) on the side of the eggs. Hundred microliter of Penicillin-Streptomycin (10,000U/ml; Thermo Fisher Scientific, MA, United States) was applied to the eggs. Then the eggs were placed under a microscope (Olympus, PA, United States). PBS (C), PBS with 40ng 1,25-dihydroxyvitamin D_3_ (1,25D-L), 200ng 1,25-dihydroxyvitamin D_3_ (1,25D-H), 40ng 25-hydroxyvitamin D_3_ (25D-L), or 200ng 25-hydroxyvitamin D_3_ (25D-H; MilliporeSigma, MO, United States) were injected into the dorsal vein of embryos (Figure 1). Afterward, glass coverslips were used to seal the windows, and injected eggs were placed back to the incubator. The injected time was recorded for individual eggs. Whole embryos were harvested at 1, 3, and 6h post-injection for gene expression analyses (eight embryos/treatment/time point). The embryos were collected in 1ml RNAlater (Thermo Fisher Scientific, MA, United States) and stored at −80C until analysis.

### RNA Extraction and Real-Time Quantitative Reverse Transcription Polymerase Chain Reaction

Total RNA was extracted from whole embryos by using QIAzol Lysis reagents (Qiagen, MD, United States) according to the manufacturer’s protocol. RNA quantity and purity were determined using a Nanodrop 1000 spectrophotometer (Thermo Fisher Scientific, Pittsburgh, PA, United States). For each sample, 2μg of RNA was reverse-transcribed to cDNA using a High-Capacity cDNA Reverse Transcription Kit (Thermo Fisher Scientific, MA, United States) following the manufacturer’s protocol in a 96-well thermal cycler (Thermo Fisher Scientific, MA, United States). cDNA templates were diluted 10-fold prior to analysis. The samples were analyzed in duplicate by real-time quantitative reverse transcription polymerase chain reaction (qRT-PCR) performed on an Applied Biosystems StepOnePlus™ (Thermo Fisher Scientific, Waltham, MA, United States) with iTaq™ Universal SYBR Green Supermix (BioRad, Hercules, CA, United States) using the following conditions for all genes: 95°C for 10min followed by 40cycles of 15s denaturation at 95°C, annealing for 20s, and 15s extension at 72°C, followed by 95°C for 15s and a melt curve stage. The key osteogenesis marker genes (*RUNX2*: runt-related transcription factor 2; *BMP2*: bone morphogenetic protein 2; *COL1A2*: collagen type I alpha 2 chain; *BGLAP*: bone gamma-carboxyglutamate protein; *SPP1*: secreted phosphoprotein 1; and *ALP*: alkaline phosphatese; Chatakun et al., 2014; Adhikari et al., 2019); key adipogenesis marker genes (*PPAR-γ*: peroxisome proliferator-activated receptor gamma; *FASN*: fatty acid synthase; and *FAPB4*: fatty acid binding protein 4; Bhat et al., 2014; Ji et al., 2015); key myogenesis marker genes (*MYOG*: myogenin; *MYOD1*: myogenic differentiation 1, and *MYF5*: myogenic factor 5; Wagatsuma and Sakuma, 2014); and a vitamin D catabolism gene (*CYP24A1*: cytochrome P450 family 24 subfamily A member 1; Christakos et al., 2010) were investigated (Table 1). *GAPDH* (glyceraldehyde-3-phosphate dehydrogenase) was used as a housekeeping gene. Samples were normalized and analyzed by the ΔΔCT method (Livak and Schmittgen, 2001).

**Table 1:** List of primers used in the current study.

Gene Name^*^	Primer sequence (5'---3')	Product size (bp)	Annealing temperature (°C)	Genome reference
*GAPDH*	GCTAAGGCTGTGGGGAAAGT TCAGCAGCAGCCTTCACTAC	161	56	NM_204305.1
*RUNX2*	TCTCTGAACTCTGCACCAAGTC GCTCGGAAGCACCTGAGAGG	229	58	NM_204128.1
*COL1A2*	CTGGTGAAAGCGGTGCTGTT CACCAGTGTCACCTCTCAGAC	222	57	NM_001079714.2
*SPP1*	GCCCAACATCAGAGCGTAGA ACGGGTGACCTCGTTGTTTT	204	57	NM_204535.4
*BMP2*	TCAGCTCAGGCCGTTGTTAG GTCATTCCACCCCACGTCAT	163	57	XM_025148488.1
*BGLAP*	GACGGCTCGGATGCTCGCAG CAGACGGGGCCGTAGAAGCG	227	57	NM_205387.3
*ALP*	CGACCACTCACACGTCTTCA CGATCTTATAGCCAGGGCCG	140	58	NM_205360.1
*PPAR-γ*	GAGCCCAAGTTTGAGTTTGC TCTTCAATGGGCTTCACATTT	131	58	XM_025154400.1
*FASN*	AGAGGCTTTGAAGCTCGGAC GGTGCCTGAATACTTGGGCT	127	58	NM_205155.3
*FABP4*	GCAGAAGTGGGATGGCAAAG GTTCGCCTTCGGATCAGTCC	153	58	NM_204290.1
*MYOD1*	GGATGCATACTACCCAGTG GCTGTCGTAGCTGTTTCT	136	58	NM_204214.2
*MYF5*	GAGAGGCAGCAGCTTCGAG GTCCACGATGCTGGAGAGG	109	58	NM_001030363.1
*MYOG*	AGCAGCCTCAACCAGCAGGA TCTGCCTGGTCATCGCTCAG	179	58	NM_204184.1
*CYP24A1*	TAATGACGGCCCTACTGCTG AGTCCTTCTGCTGCGCTAAA	271	58	NM_204979.1

* *GAPDH*, glyceraldehyde-3-phosphate dehydrogenase; *RUNX2*, runt related transcription factor 2; *COL1A2*, collagen type I alpha 2 chain; *SPP1*, secreted phosphoprotein 1; *BMP2*, bone morphogenetic protein 2; *BGLAP*, bone gamma-carboxyglutamate protein; ALP, alkaline phosphatase; *PPAR-γ*, peroxisome proliferator-activated receptor gamma; *FASN*, Fatty acid synthase; *FABP4*, fatty acid binding protein 4; *MYOD1*, myogenic differentiation 1; *MYF5*, myogenic factor 5; *MYOG*, myogenin; *CYP24A1*, cytochrome P450 family 24 subfamily A member 1.

### Statistics

All experimental data were analyzed statistically by one-way ANOVA using SAS software Version 9.3 (SAS Institute, Cary, NC). Variability in the data was expressed as standard error mean (SEM). Differences between means were determined using Duncan’s Multiple Range test. The level of significance was assessed at *p*≤0.05.

## Results

The sequence for *CYP27B1* (encoding 1α-hydroxylase) has not yet been identified in chickens (NCBI). 1α-hydroxylase is the critical enzyme that catalyzes 25OHD to 1,25OHD (Christakos et al., 2010). Thus, its expression to indicate the conversion of 25OHD to 1,25OHD could not be examined in the current study. Additionally, the level of 1,25OHD is difficult to measure in the embryo, because the sampling of embryo could not avoid breaking blood vessels, which could convolute analysis. However, the expression of *CYP24A1* encoding 24-hydroxylase that is responsible for 1,25OHD inactivation to 1,25,24 OHD (van Driel and van Leeuwen, 2014) could be stimulated by 1,25OHD (Veldurthy et al., 2016). Thus, by investigating *CYP24A1* expression level, we could observe the 1,25OHD residues status in the embryos.

**Figure 2:**
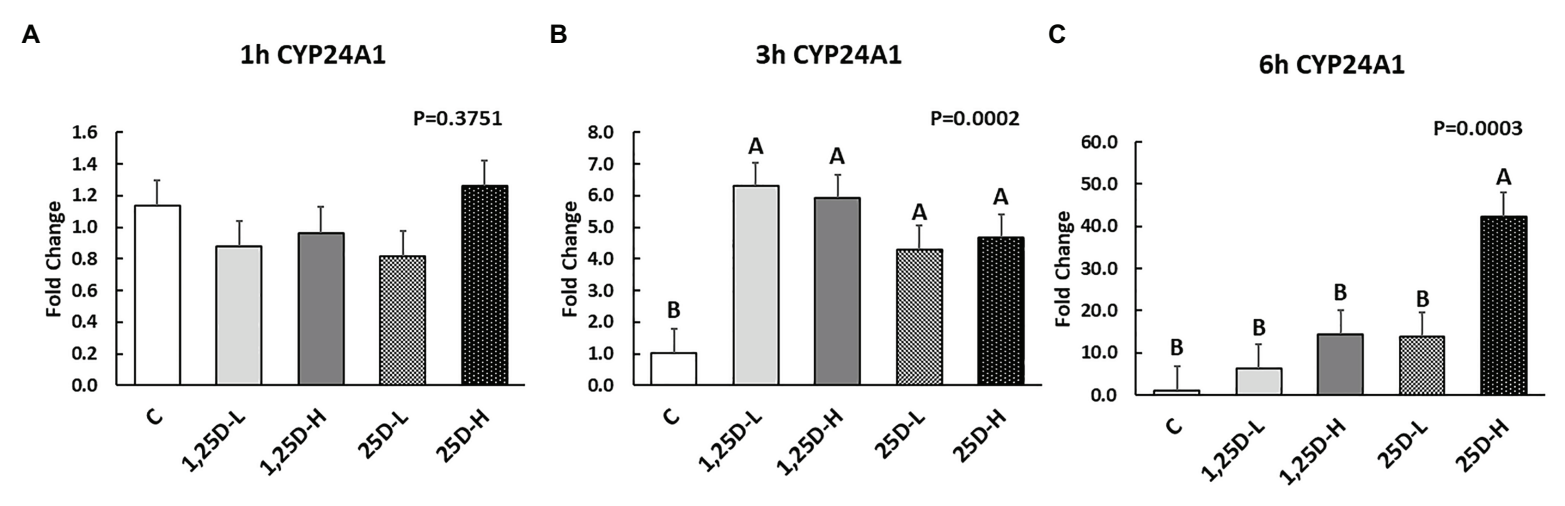
Effect of 25OHD and 1,25OHD on *CYP24A1* expression at 1h **(A)**, 3h **(B)**, and 6h **(C)** post injection. Mean separation was indicated by different letters on the top of bars (value means±SEM, *n*=8). *CYP24A1*: cytochrome P450 family 24 subfamily A member 1.

**Figure 3:**
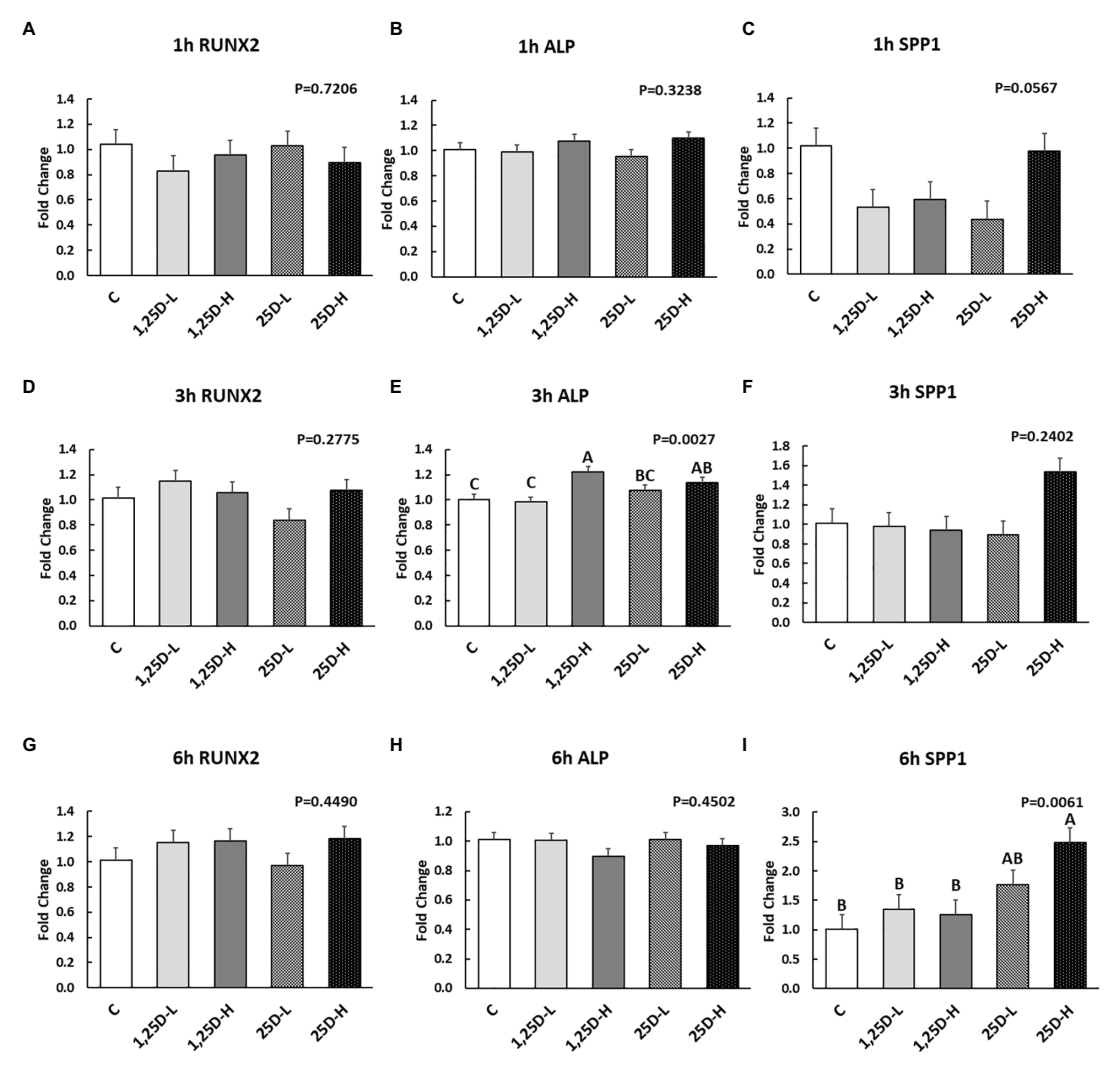
Effect of 25OHD and 1,25OHD on key osteogenesis marker gene expressions. **(A–I)** showed the expression of *RUNX2*, *ALP* and *SPP1* at 1h, 3h, and 6h post-injection, respectively. Mean separation was indicated by different letters on the top of bars (value means±SEM, *n*=8). *RUNX2*: runt related transcription factor 2; *ALP*: alkaline phosphatase; *SPP1*: secreted phosphoprotein 1.

**Figure 4:**
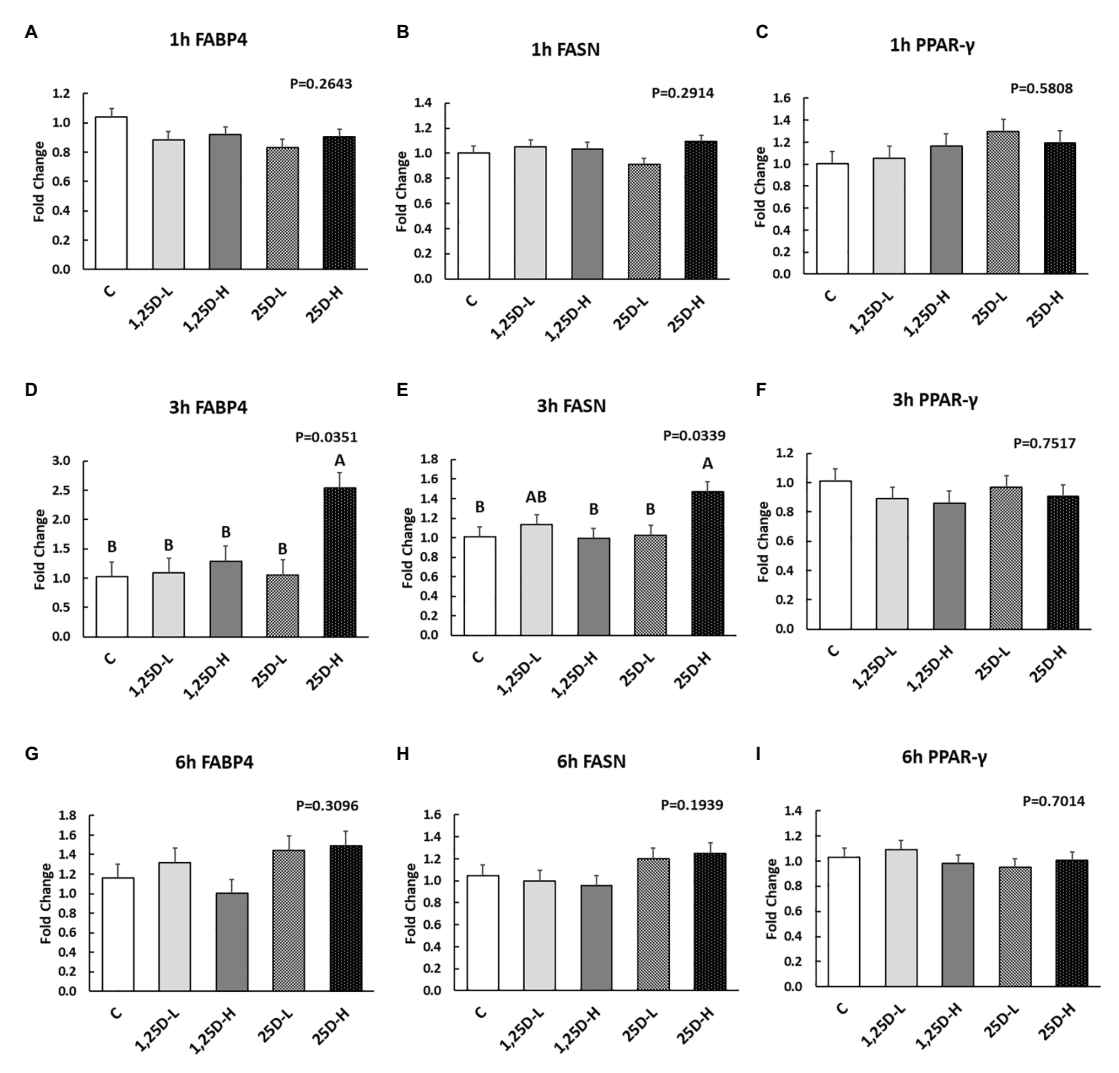
Effect of 25OHD and 1,25OHD on key adipogenesis marker gene expressions. **(A–I)** showed the expression of *FABP4*, *FASN* and *PPAR-γ* at 1h, 3h, and 6h post-injection, respectively. Mean separation was indicated by different letters on the top of bars (value means±SEM, *n*=8). *FASN*: fatty acid synthase; *FABP4*: fatty acid binding protein 4; *PPAR-γ*: peroxisome proliferator-activated receptor gamma.

**Figure 5:**
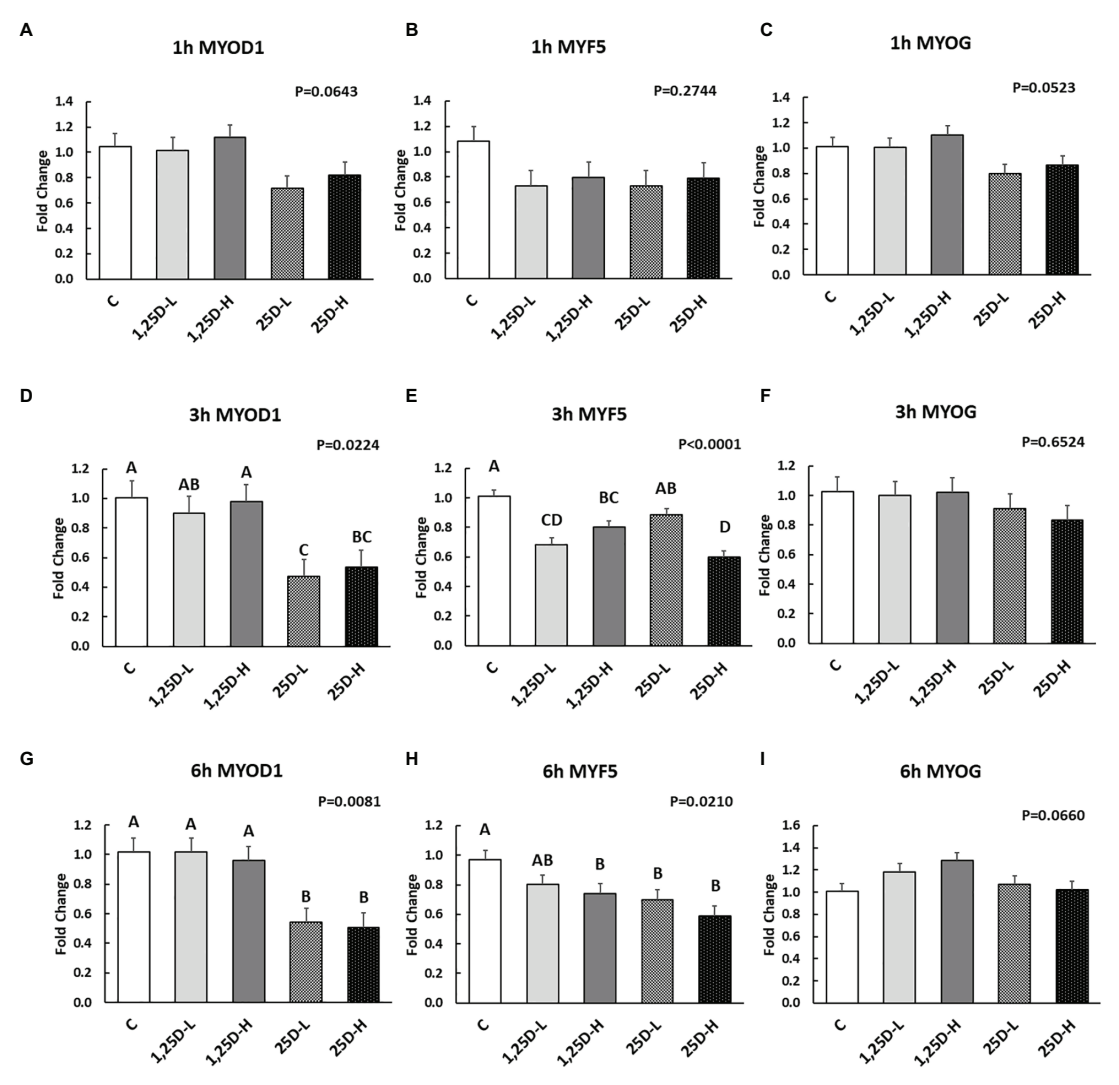
Effect of 25OHD and 1,25OHD on key myogenesis marker gene expressions. **(A–I)** showed the expression of *MYOD1*, *MYF5* and *MYOG* at 1h, 3h, and 6h post-injection, respectively. Mean separation was indicated by different letters on the top of bars (value means±SEM, *n*=8). *MYOD1*: myogenic differentiation 1; *MYF5*: myogenic factor 5; *MYOG*: myogenin.

Since no differences were observed in *BMP2*, *COL1A2*, and *BGLAP* expression, these data were not shown in this manuscript. At 1h post-injection, there was no difference in the vitamin D_3_ metabolism marker (*CYP24A1*) expression (Figure 2A). Meanwhile, no significant differences were observed on osteogenesis (Figures 3A–C), adipogenesis (Figures 4A–C), and myogenesis marker gene expression (Figures 5A–C).

At 3h post-injection, all treatments showed higher *CYP24A1* expression than control (*p*=0.002; Figure 2B), which indicated the injected 1,25OHD and 25OHD may have activated the catabolism of vitamin D_3_ in the embryos. At the same time, early osteogenesis differentiation marker (*ALP*) was increased by high 1,25OHD and high 25OHD injection compared to the control (*p*=0.0027; Figure 3E). However, no differences were found in *RUNX2* (Figure 3D) and *SPP1* expression (Figure 3F). With respect to genes involved in adipogenesis, high 25OHD injection induced the expression of *FABP4* (*p*=0.0351; Figure 4D) and *FASN* (*p*=0.0339; Figure 4E) compared to the control group, although no differences were detected in the expression of *PPAR-γ* (Figure 4F). Meanwhile, clear inhibitory effects of vitamin D injection on expression of genes regulating myogenesis were observed. 25OHD treatments significantly decreased *MYOD1* expression at 3h (*p*=0.0224; Figure 5D), and 1,25OHD treatments, and high level 25OHD suppressed the expression of *MYF5* (*p*<0.0001; Figure 5E). No difference in *MYOG* expression was observed among treatments (Figure 5F).

At 6h post-injection, *CYP24A1* expression was significantly higher in 25D-H (*p*=0.0003; Figure 2C), indicating a higher dosage of 25OHD possibly obtained a longer retention time. Meanwhile, 25D-H has higher *SPP1* expression compared to the control (*p*=0.0061; Figure 3I), but no difference was found in *RUNX2* and *ALP* expression (Figures 3G,H). There were also no differences in adipogenesis gene expression (Figures 4G–I). However, 25OHD treatments down-regulated *MYOD1* expression (*p*=0.0008; Figure 5G), and both high 1,25OHD and 25OHD treatments suppressed *MYF5* expression (*p*=0.0210; Figure 5H). Similar to other time points, no difference in *MYOG* expression were identified at 6h post-injection (Figure 5I).

## Discussion

In the current study, 25OHD and 1,25OHD showed similar effects on embryogenesis, which indicated the embryo might have acquired the ability to convert 25OHD to 1,25OHD at ED 3. However, to our knowledge, the earliest time that the kidney of chicken embryos can produce 1,25OHD is reported at ED 9 (Bishop and Norman, 1975). Nevertheless, the mesonephros (intermediate kidney) begins to appear at about 55h of incubation, and fully formed at ED 4 (Sturkie, 2012; Bolin and Burggren, 2013). It suggested that the intermediate kidney at this embryo stage could convert 25OHD to 1,25OHD (Kubota et al., 1981). Moreover, *CYP27B1* (1α-hydroxylase) has reportedly been expressed in lymph nodes, skin, colon, pancreas, dendritic cells, brain, pulmonary alveolar macrophages, pathological parathyroid glands, prostate cells, and bones in various animal models (Panda et al., 2001; Zehnder et al., 2001; van Driel et al., 2006; Atkins et al., 2007; Adams and Hewison, 2012), suggesting that 25OHD may be converted by tissues outside of the kidney. A previous study has shown that 25OHD could inhibit the proliferation of myogenic cells in a similar manner to 1,25OHD, suggesting that the conversion of 25OHD to 1,25OHD may happen in myoblasts (Srikuea et al., 2012). However, further research needs to be conducted to validate the earliest time that chicken embryos can metabolize vitamin D_3_.

Both 1,25OHD and 25OHD are metabolized by *CYP24A1* (24-hydroxylase), becoming 1,24,25OHD and 24,25OHD, respectively (Norman et al., 1980). 1,25OHD induces *CYP24A1* synthesis *via* several pathways to form a negative feedback loop, avoiding excess 1,25OHD formation (Zierold et al., 1995; DeLuca, 2004). Thus, the expression of *CYP24A1* could indicate the catabolism status of vitamin D_3_ metabolites. Results indicated that the injection had not triggered the enhanced catabolism process at 1h post-injection. However, at 3h post-injection, all the treatments showed higher *CYP24A1* expression compared to control group. At 6h post-injection, only the higher level of 25OHD obtained the highest expression of *CYP24A1*. The catabolism of vitamin D_3_ in the embryos was surprisingly fast, which may be attributed to the fact that catabolism of vitamin D_3_ is tightly and rapidly regulated by 1,25OHD (Haussler et al., 2013).

The current study showed the positive effects of vitamin D_3_ metabolites on *ALP* (alkaline phosphatase) and *SPP1* (osteopontin) expression at 3 and 6h post-injection, respectively. *ALP* is an early osteogenesis marker and is essential for bone mineralization by enhancing the initial mineral crystal formation (Chatakun et al., 2014). *SPP1* codes a highly phosphorylated sialoprotein with strong mineral-binding capacities in the extracellular matrix (Chabas, 2005). The role of vitamin D_3_ metabolites on osteogenesis has been extensively investigated. However, cell culture studies on the influence of vitamin D_3_ on osteogenesis have produced varied, and at times conflicting, results (van Driel and van Leeuwen, 2014). In agreement with our findings, 1,25OHD has been shown to stimulate on *ALP* expression in human osteoblasts (Matsumoto et al., 1991; Siggelkow et al., 1999; Chen et al., 2002; Woeckel et al., 2010). However, 1,25OHD is also reported to down-regulate *ALP* expression in mouse osteoblasts (Chen et al., 2012; Kim et al., 2016). Meanwhile, expression of *Spp1* has been reported to increase in response to 1,25OHD in ROS 17/2.8 cell (rat; Staal et al., 1996). These inconsistent results may reflect different experimental conditions such as species, cell stage, treatment time, and dosages (van Driel and van Leeuwen, 2014). Besides the *in vitro* studies’ contradictory results, additional vitamin D_3_ or 25OHD injected to chicken embryos at ED 18 revealed a positive effect on bone quality of hatched chicks (Abbasi et al., 2017; Zamani et al., 2018). It is important to note that the cell culture system has limitations such as missing interactions of different types of cell and lacks consideration of other extracellular factors (van Driel and van Leeuwen, 2014). Indeed, it has been reported that factors, such as phosphate concentration, growth factors, and cytokines, may affect the function of 1,25OHD (van Driel and van Leeuwen, 2014).

The stimulatory effects of vitamin D_3_ metabolites on adipogenesis at 3h post-injection were observed in the current study. However, in previous studies, the effects of vitamin D_3_ metabolites on adipocyte differentiation were inconsistent. Stimulatory effects were found in murine studies, but inhibitory effects were frequently shown in the humans (Dix et al., 2018). Other animal studies showed mixed results and are thoroughly reviewed in Dix et al. (2018). In chickens, an *in vivo* study showed that feeding additional 25OHD did not change the fat pad weight but did increase monounsaturated fatty acids and reduced the polyunsaturated fatty acids (Michalczuk et al., 2010). Even though the vitamin D_3_ interactions with adipose tissue have been reported in various animal and human models (Ding et al., 2012), limited data are available on vitamin D_3_ metabolites and chicken adipocytes. The research herein may contribute to our understanding of the fatty liver in old laying hens.

Both osteoblasts and adipocytes are differentiated from embryonic stem cells and mesenchymal stem cells (Kolf et al., 2007). A study has shown that PPAR-γ, the master regulator of adipogenesis (Kawai and Rosen, 2010), could alter mesenchymal stem cells (MSCs) fate by suppressing osteogenic transcription factors, such as homeobox protein DLX5, RUNX2, and Osterix (Kawai and Rosen, 2010), indicating a reciprocal relationship between osteogenic and adipogenic differentiation. Similar results were found in the previous study, where 1,25OHD increased adipogenic differentiation but inhibited osteoblastic cell proliferation and differentiation in rat bone marrow stromal cells (Atmani et al., 2003). However, in the current study, vitamin D_3_ metabolites stimulated osteogenesis and adipogenesis simultaneously, evidenced by increased expression of *FAPB4* and *FASN* (adipogenesis markers), and *ALP* (osteogenesis marker) at 3h post-injection. This may reflect that the chicken embryo contains a number of extracellular factors, such as fibroblast growth factors, insulin, and growth factors, contributing to the complexity of vitamin D_3_ metabolism on adipogenesis (Rosen and MacDougald, 2006).

In the current study, the inhibitory effects of 1,25OHD on myogenic differentiation were shown as a decrease of *MYOD1* and *MYF5* expression at both 3 and 6h post-injection. *MYF5* and *MYOD1* are families of Helix-Loop-Helix transcription factors that are expressed during myoblast proliferation and myotube differentiation (Bismuth and Relaix, 2010; Braun and Gautel, 2011). In agreement with the current study, VDR (vitamin D receptor) knockout mice had higher Myf5 and MyoG expression compared to the normal mice (Endo et al., 2003), which indicated the regulatory effects of vitamin D_3_ on myoblast differentiation. On the contrary, it has been reported that 1,25OHD has a stimulatory effect on the proliferation and differentiation of embryonic chick myoblasts in culture, suggested by an increase in both cell density and fusion after 1,25OHD treatment (Giuliani and Boland, 1984). Likewise, in mice, treatment of C2C12 (mouse myoblast) cells with 1,25OHD increased the *MyoD1* and *MyoG* expression (Garcia et al., 2011). The current paper is contributing to the understanding of these species/cell specific data as it has its own set of unique variables and context. However, the specific pathways involved in such an effect in chicken embryo need to be investigated further.

In summary, 25OHD and 1,25OHD administration to embryos elicited similar responses, suggesting that the embryo may be able to convert 25OHD to 1,25OHD; however, further research is necessary to determine the specific tissue location of the conversion. The catabolism of injected vitamin D_3_ metabolites appeared to be remarkably fast based on the expression of a key vitamin D_3_ catabolism-related gene (*CYP24A1*). The higher dosage of 25OHD showed a possibility of a longer retention time in the embryo. Additionally, both 1,25OHD and 25OHD increased the expression of osteogenesis and adipogenesis-related genes but inhibited myogenesis-related gene expressions during early embryo development in this study. However, the detailed pathways involved in these effects need further studies. Our findings provide an overview of the role of vitamin D_3_ metabolites in early chicken embryogenesis and the potential basis of practical strategies of early nutrient supplementation in chicken embryos.

## Data Availability Statement

The original contributions presented in the study are included in the article/supplementary material, further inquiries can be directed to the corresponding author.

## Ethics Statement

The animal study was reviewed and approved by the Institutional Animal Care and Use Committee at the University of Georgia.

## Author Contributions

CC and DW: investigation. WK and CC: validation and data curation. CC: formal analysis, writing – original draft preparation, and visualization. WK, BM, and CC: writing – review and editing. WK: supervision, project administration, and funding acquisition. All authors contributed to the article and approved the submitted version.

### Conflict of Interest

The authors declare that the research was conducted in the absence of any commercial or financial relationships that could be construed as a potential conflict of interest.
